# Effects of problem-solving interventions on aggressive behaviours among primary school pupils in Ibadan, Nigeria

**DOI:** 10.1186/s13034-016-0116-5

**Published:** 2016-09-02

**Authors:** Jibril Abdulmalik, Cornelius Ani, Ademola J. Ajuwon, Olayinka Omigbodun

**Affiliations:** 1 Department of Psychiatry, College of Medicine, University of Ibadan & University College Hospital, Ibadan, Nigeria; 2 Centre for Child and Adolescent Mental Health, University of Ibadan, Ibadan, Nigeria; 3 Centre for Mental Health, Imperial College London, London, UK; 4 Department of Health Promotion and Education, College of Medicine, University of Ibadan, Ibadan, Nigeria

**Keywords:** Problem solving skills, Interventions, Aggressive behaviors, Students, Nigeria

## Abstract

**Background:**

Aggressive patterns of behavior often start early in childhood, and tend to remain stable into adulthood. The negative consequences include poor academic performance, disciplinary problems and encounters with the juvenile justice system. Early school intervention programs can alter this trajectory for aggressive children. However, there are no studies evaluating the feasibility of such interventions in Africa. This study therefore, assessed the effect of group-based problem-solving interventions on aggressive behaviors among primary school pupils in Ibadan, Nigeria.

**Methods:**

This was an intervention study with treatment and wait-list control groups. Two public primary schools in Ibadan Nigeria were randomly allocated to an intervention group and a waiting list control group. Teachers rated male Primary five pupils in the two schools on aggressive behaviors and the top 20 highest scorers in each school were selected. Pupils in the intervention school received 6 twice-weekly sessions of group-based intervention, which included problem-solving skills, calming techniques and attribution retraining. Outcome measures were; teacher rated aggressive behaviour (TRAB), self-rated aggression scale (SRAS), strengths and difficulties questionnaire (SDQ), attitude towards aggression questionnaire (ATAQ), and social cognition and attribution scale (SCAS).

**Results:**

The participants were aged 12 years (SD = 1.2, range 9–14 years). Both groups had similar socio-demographic backgrounds and baseline measures of aggressive behaviors. Controlling for baseline scores, the intervention group had significantly lower scores on TRAB and SRAS 1-week post intervention with large Cohen’s effect sizes of 1.2 and 0.9 respectively. The other outcome measures were not significantly different between the groups post-intervention.

**Conclusions:**

Group-based problem solving intervention for aggressive behaviors among primary school students showed significant reductions in both teachers’ and students’ rated aggressive behaviours with large effect sizes. However, this was a small exploratory trial whose findings may not be generalizable, but it demonstrates that psychological interventions for children with high levels of aggressive behaviour are feasible and potentially effective in Nigeria.

## Background

Aggressive behaviors among young people represents a wide spectrum that ranges from a major public health concern [1, 2]; to difficulties with academic performance, school underachievement, disciplinary problems, high drop-out rates, psychoactive substance use and getting into trouble with the law [3]. The World Health Organization (WHO) estimates that interpersonal violence among young people below the age of 19 years accounts for 227 deaths daily [1]. Many more individuals suffer from injuries and traumatic experiences arising from violence and aggressive behaviors [2]. Once a pattern of aggressive behavior is established in childhood, it often persists into adulthood with attendant negative consequences [4, 5]. A longitudinal study of developmental outcomes reported that children with high aggressive behaviours were 2.4 times more likely to exhibit disruptive behaviours (CI 2.1–5.1); 3.3 times more likely to be male (CI 2.1–5.1); and 2.9 times more likely to have substance abuse/dependence problems (CI 1.9–4.5) in adulthood [5]. A high level of physical aggression in childhood is also strongly predictive of future criminality [6]. Aggressive behaviours in early childhood have also been shown to be a more consistent predictor of poor social functioning than inattention, hyperactive-impulsive or oppositional behavior [7]. In the short term, aggressive children are more likely to be disruptive in school, bully their peers, and be excluded from schools [8]. Thus, early identification of children with aggressive behaviours may be particularly important to prevent social difficulties and improve long-term outcomes [6, 7]. Boys are 5 times more likely to exhibit high levels of physical aggression than girls [9, 10].

Schools are the most important settings outside the home, where a child’s views, attitudes and behaviors are shaped early in life [11]. This makes the school environment a good setting for identifying and providing targeted early intervention for children with high levels of aggressive behaviors. Several early intervention programs using parent training, social skills training for children and teacher support (singly or in combination) have demonstrated good outcomes [8, 10, 12, 13]. A meta-analysis of school-based interventions for aggressive and disruptive behaviours found that the most successful improvements occurred when the intervention was focused on students with the highest risk of aggressive behaviors [8, 10]. Hostile attributional bias predicts reactive aggressive behaviours in children [14]; and interventions such as those focusing on social and emotional learning have demonstrated effectiveness in reducing aggressive behaviours, while improving prosocial ratings [15]. Group based interventions have also been found to be effective in reducing externalizing behaviours among children in school settings [16].

However, the majority of these intervention studies come from developed countries, especially the United States of America (USA) and Canada. To our knowledge, there are no published school-based interventions studies against aggression from Africa. Given the huge cultural, social, and demographic differences between developed countries and low and middle income countries (LMICs) like Nigeria, it cannot be assumed that interventions against aggression that are effective in developed countries would be equally useful in settings such as Nigeria. LMICs are characterized by insufficient numbers of mental health professionals, and reduced access to mental health care services; all of which culminate in a huge treatment gap [17, 18]. Furthermore, some persisting cultural child rearing practices in parts of Nigeria, appear to expose the child to aggressive patterns of behavior—both in the home and on the streets, as well as the routine utilization of punitive measures for child discipline [19–22]. An alternative, non-punitive intervention for children with high levels of aggressive behaviours could potentially be a useful recommendation for widespread uptake. Such interventions are particularly relevant for schools in LMICs such as Nigeria, which has an average primary school net enrollment ratio (NER) of 66 %; and an average secondary school NER of 27 %. Thus, every effort to ensure children who attend school are retained in school and not allowed to drop out or fall through the cracks is of vital importance [23]. This study therefore aimed to assess the feasibility and effectiveness of a group-based problem-solving intervention for primary school pupils with high levels of aggressive behaviors in Ibadan, Nigeria. The views of the class teachers about causes of aggressive behaviours as well as possible strategies for reducing such behaviours were also assessed.

## Methods

### Study design

This was an intervention study with a treatment and a wait-list control groups. Two public primary schools in the Bere neighborhood of Ibadan North East Local Government Area with similar profiles were selected and randomly allocated to an intervention or control arm. This area was selected due to its high-density urban population, and its lower socio-economic status with a lack of basic social amenities such as potable water. The area is also noted for its high rates of violence and aggression, which may be mirrored by the children growing up in such neighbourhoods. Children attending primary school education in the study setting usually enroll in primary one at an average age of 6 years and complete the 6 years of primary education averagely by the age of 12 years. The intervention and control schools had average class sizes of 52 and 50, with two teachers assigned to each class. The schools did not have student counsellors or formal behavioral management programs. At the time of the study, culturally approved corporal punishment was the most commonly utilized disciplinary strategy used by teachers in both schools.

### Participants and recruitment

**Fig. 1:**
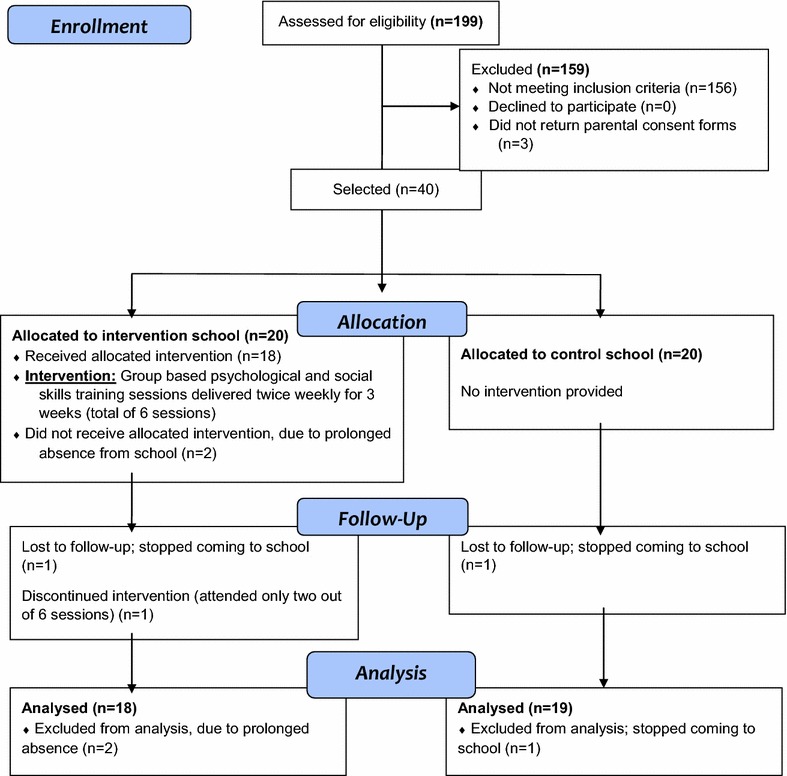
Consort flow chart summary of study participants

The subjects were male students in primary five. Males were selected because of the clear evidence that they are more likely to engage in physically aggressive behaviours than females [9, 10]. A more senior class (primary five) was selected to ensure that the children would be developmentally mature enough to understand and utilize the cognitive problem-solving skills contained in the intervention. The class teachers rated all male primary five students whose parents consented, on their levels of aggressive behaviours. The top 20 highest scoring students were selected to ensure that the students with the greatest need participated in the intervention. Eligible students with a poor understanding of the local Yoruba language (ascertained either by self-admission, or by interactions using the Yoruba language); as well as those with probable learning disability (identified by the class teachers as have significant learning difficulties) were excluded and replaced by the next eligible student on the list. Using techniques described by Wade [24], a sample size of 16 (for each group) was calculated apriori as adequate to identify a reduction of one standard deviation in aggressiveness in the intervention group compared with the control group based on 80 % power and 5 % level of significance. This was increased to 20 in each group to account for possible attrition in the course of the study. Eighteen students in the treatment group completed the intervention and 19 students in the control arm completed post treatment assessment. The students completed the assessments anonymously; as their names were not utilized and they were assured that their responses would be confidentially handled and not reported to their teachers or parents. Figure 1 shows the case flow.

### Study instruments

1. A socio-demographic questionnaire.
  This obtained information on age, family characteristics such as size and structure, and their ownership of valued household items such as mobile phones, television, refrigerator, motorcycle, car, and satellite dish. These latter items were used to assess socio-economic status.
2. Teacher rating of students’ aggressive behaviours (TRAB).
  This 15-item questionnaire was adapted from two previous studies [25, 26]. The questionnaire sought teachers ‘views on each student’s involvement in common examples of overt aggressive behaviours such as frequently taunting, threatening or initiating fights with other children in school in the previous month. Responses were rated on a 3 or 5 point Likert scale. The 3 point Likert scale options were rated as: *not true* (0); *sometimes true* (1); and *often true* (2). The 5 point Likert scale questions were rated as *never true* (0); *rarely true* (1); *sometimes true* (2); *usually true* (3); and *almost always true* (4). The total score ranged from 0 to 42, with higher scores indicating more aggressive behaviours.
3. Teacher rated strengths and difficulties questionnaire (SDQ).
  The SDQ is a 25 item screening questionnaire for emotional and behavioural problems in children and adolescents [27]. The SDQ is a well-validated and reliable instrument, which has been used successfully in Nigeria [28] and many other developing countries [29, 30]. The SDQ has five subscales—emotional, conduct, hyperactivity, peer problems and prosocial. A “total difficulties score” is derived from the sum of the first four subscales, ranging from 0 to 40, with higher scores indicating greater difficulties.
4. Self-rated aggression scale (SRAS).
  The SRAS is a self-completed 14-item questionnaire that has been used successfully in a previous study in Nigeria [31]. Students indicated on a 3 point Likert scale whether they have been involved in various types of aggressive behaviours such as hitting, name calling, and teasing in the past 3 weeks. Sample questions include: ‘*Did you slap or kick someone?*’; ‘*Did you threaten to hurt or hit someone?*’; and ‘*Were you involved in a physical fight because you were angry?*’. These items were scored as ‘*not true*’ (0); ‘*sometimes true*’ (1); and ‘*very true*’ (2). Total scores ranged 0–14, with higher scores indicative of more self-rated aggressive behaviours. Information was also sought on whether they have ever used a weapon, been injured or injured someone else in a fight, and if they belonged to a gang.
5. Attitude towards aggression questionnaire (ATAQ).
  This questionnaire consisted of (a) four items that sought student’s views on the appropriateness of retaliation with aggression, (b) eight items to assess their attitude to statements that support aggression, and (c) six items on how they would cope with various situations that could provoke aggression. These were rated on 4-point Likert scale and summed such that higher scores indicate more favourable attitude towards aggression. The items were adapted from previous studies [32, 33]. Sample items include: ‘*it’s ok to get into physical fights with others if they make you angry*’; ‘*sometimes, you have to hit another child, if you think they are going to hit you first*’; ‘*if another boy wants to fight with me, it is better to talk to him than to fight*’; ‘*if you refuse to fight, everyone will think you are a weak coward*’. The options range from ‘*strongly disagree*’ (1), ‘*disagree*’ (2), ‘*agree*’ (3) to ‘*strongly agree*’ (4). Total scores range from 19 to 76, with higher scores indicating higher propensity towards aggressive behaviours.
6. Social cognition and attribution scale (SCAS).
  This questionnaire assessed the students’ attributional styles in ten hypothetical scenarios demonstrating ambiguous peer intent [30, 34]. For each scenario, students were asked to what extent the hypothetical peer’s behaviour was likely to have been on purpose or by accident. They were also asked to rate on a 3-point scale how they were likely to have reacted if they had in fact been involved in a similar situation. Both their attribution of intent and likely reactions were summed, with total scores ranging from 0 to 28; and higher scores indicated more hostile attribution and more aggressive response respectively. A sample scenario is presented here: *‘If you are on the playground and someone pushes you down when you were not looking, how will you think it happened?*’The options are: (*a*)*. it was an accident* and (*b*)*. it was intentional*. The follow up question then specifies: *‘If this happened to you, what will you do?’* Options will be: (*a*)*. I will hit him;* (*b*)*. I will ask him why he pushed me down;* and (*c*)*. I will tell him it’s okay, it was an accident.*

### The intervention

The thinking group (problem solving intervention protocol) manual was adapted by the second author from the Brain Power Program [8]. The manual was further developed with field-testing by the first author. It is a group-based problem solving skills and attributional retraining program for aggressive students. The manual included scenarios and examples that were contextualized for the Nigerian environment. Examples include using locally relevant scenarios that the children can easily relate with, such as the warnings from a referee in a football game (which is the most popular game among boys and male adults in Nigeria). Thus, in explaining the principle of STOP, THINK before ACTING (STA); the analogy of traffic lights (red for Stop, amber for Think and green for Act) was replaced by the referee STOPPING the game for a foul, handing out a yellow card (THINK), and the player subsequently ACTING properly and carefully to avoid a red card (eviction from the game). The intervention was translated into the local Yoruba language and delivered by a clinical psychologist who is a fluent native Yoruba speaker. The first author who is also fluent in Yoruba supervised the psychologist on-site. Each session included 10 students and utilized an interactive workshop-format lasting 40 min.

The first session introduced the programme and worked on motivational strategies to help the students engage with the rest of the programme. The second session taught the students calming techniques such as calming self-talk and deep slow breathing. Session three covered problem-solving strategies while sessions four and five focused on attribution retraining. These latter sessions taught the students how to distinguish between willful and accidental intent, and recognize ambiguity in interpersonal interactions. The sixth session was utilized to recap the salient points in all previous sessions. This skill-based training was delivered twice weekly for 3 weeks.

#### Teachers assessment

The class teachers in the intervention school were invited to observe the sessions unobtrusively. Their views were sought pre and post intervention on (a) possible triggers of aggression, (b) strategies to manage aggression, and (c) their attitude towards psychologically based intervention for aggressive children.

#### Study procedure

The TRAB questionnaires were dropped for the class teachers in the two schools to rate all the children in their class who had parental consent, and had assented to participate in advance. The TRAB scores were utilized to identify the top 20 boys with the highest teacher-rated scores for aggressive behaviours. These students were subsequently recruited into the study, and study measures were completed at baseline in both schools. The participants in the intervention school received six sessions of the intervention, delivered twice weekly for 3 weeks. It was delivered as a group-based intervention in small groups of 10 boys in each group. The participants in the waiting-list control school did not receive any intervention. Afterwards, the study measures were repeated again in both schools.

Class teachers in the intervention school were invited to witness at least two sessions, unobtrusively as quiet observers seated at the back of the hall. They were simply to observe and did not participate at all, in order to avoid disrupting the group dynamics.

### Data management

Data was analysed with SPSS Version 21. Continuous univariate data such as age and scores on rating scales are described with means and standard deviations while categorical variables are described as proportions and frequencies. Bivariate comparisons s between the intervention and control groups were conducted with student t tests for normally distributed continuous variables and Chi square for categorical variables. Treatment effect was assessed with analysis of covariance (ANCOVA) of post-treatment scores controlling for baseline scores. Cohen’s effect sizes were calculated with 0.2, 0.5, and 0.8 considered by convention as small, medium, and large respectively [35]. In view of the relatively large number of outcome measures involving multiple comparisons, the data was statistically restricted with Bonferonni adjustment with significance level set at 0.01.

## Results

### Socio-demographic profile of respondents

The students ranged in age from 9 to 14 years (Mean 12 years, SD 1.27). The two groups did not differ significantly in their socio-demographic characteristics (Table 1) or baseline scores on the outcome measures except the SDQ conduct subscale, where the intervention group scored higher than controls, although this was not significant (p = 0.24).

**Table 1:** Comparison of baseline demographic variables for the two groups

No	Variable	Treatment group (N = 20)	Control group (N = 20)	Test t (df) or X^2^	p value
1	Age (mean, SD)	12.28 (1.07)	11.89 (1.45)	−0.91 (35)	0.37
2	Number of mother’s children (mean, SD)	4.50 (1.89)	4.63 (1.80)	0.22 (35)	0.83
3	Number of rooms in the home (mean, SD)	1.33 (0.49)	1.21 (0.42)	−0.25 (35)	0.42
4	Number of people living in the house (mean, SD)	5.11 (1.97)	5.74 (2.85)	0.77 (35	0.44
5	Family type: n (%)				
	Monogamous	11 (61.1)	14 (73.7)	0.67	0.50
	Polygamous	7 (38.9)	5 (26.3)		
6	Parents’ status: n (%)				
	Living together	12 (66.7)	13 (68.4)	0.01	1.0
	Separated/late	6 (33.3)	6 (31.6)		
7	Valued household items: n (%)				
	Less than 3 items	3 (16.7)	5 (26.3)	0.51	0.69
	3 or more items	15 (83.3)	14 (73.7)		
8	Academic performance n (%)				
	Top half of the class	11 (61.1)	14 (73.7)	1.49	0.34
	Bottom half of the class	7 (38.9)	5 (26.3)		

### Effectiveness of intervention

The pre- and post-intervention scores on outcome variables for the treatment and control groups are presented in Tables 2 and 3 respectively. Statistically significant reductions in the post intervention scores were observed for the TRAB, SRAS and all three components of the ATAQ in the intervention group; whereas for the control group, the post intervention scores only showed a significant reduction in the TRAB and Coping strategies component of the ATAQ, while the SRAS scores increased.

**Table 2:** Comparison of pre and post intervention scores on outcome measures for the experimental group (N = 18)

No	Variable	Pre-intervention	Post-intervention	t test t (df)	p value
1	Total TRAB (mean, SD) [range of 0–42]	29.6 (6.9)	18.2 (8.6)	5.18 (17)	<0.001*
2	SDQ (mean, SD)				
	Emotional	3.9 (1.5)	4.6 (1.7)	−1.23 (17)	0.24
	Conduct	6.17 (2.5)	6.0 (3.4)	0.24 (17)	0.81
	Hyperactivity	5.9 (2.4)	7.6 (4.9)	−1.40 (17)	0.18
	Peer problems	5.5 (2.0)	6.3 (2.7)	−1.05 (17)	0.31
	Total difficulties score [range of 0–40]	21.5 (5.6)	24.4 (7.8)	−1.35 (17)	0.19
	Prosocial	7.7 (3.8)	6.8 (3.9)	1.76 (17)	0.10
3	SRAS (mean, SD) [range of 0–14]	8.7 (4.3)	6.1 (4.9)	2.47 (17)	0.02*
4	ATAQ (mean, SD) [range of 19–76]				
	Retaliation belief	9.4 (3.3)	5.8 (2.7)	5.37 (17)	<0.001*
	General belief	21.8 (5.5)	17.2 (5.5)	4.14 (17)	0.001*
	Coping strategies	16.8 (3.1)	22.1 (2.8)	−4.42 (17)	<0.001*
5	SCAS (mean, SD) [range of 0–28]	16.2 (6.4)	13.8 (5.3)	1.79 (17)	0.09

Two students dropped out

*TRAB* Teacher rating of aggressive behaviours (total maximum score = 42), *SDQ* Strengths and Difficulties Questionnaire (total maximum score = 40), *SRAS* Self rated aggression scale (total maximum score = 14), *ATAQ* Attitude towards aggression questionnaire (total maximum score = 76), *SCAS* Social cognition and attribution scale (total maximum score = 28)

* Data was statistically restricted with Bonferonni adjustment

**Table 3:** Comparison of pre and post intervention scores on outcome measures for the control group (N = 19)

No	Variable	Pre-intervention	Post-intervention	t test t (df)	p value
1	Total TRAB (mean, SD) [range of 0–42]	32.4 (5.7)	26.5 (4.9)	3.12 (18)	0.006*
2	SDQ (mean, SD)				
	Emotional	4.3 (2.2)	5.7 (4.9)	−1.41 (18)	0.18
	Conduct	4.4 (2.1)	4.0 (1.5)	0.97 (18)	0.35
	Hyperactivity	5.3 (1.3)	5.8 (2.0)	−0.85 (18)	0.41
	Peer problems	5.8 (1.4)	6.9 (3.2)	−1.57 (18)	0.13
	Total difficulties score [range of 0–40]	19.7 (4.4)	22.4 (7.5)	−1.62 (18)	0.12
	Prosocial	6.4 (2.2)	6.7 (1.4)	−0.67 (18)	0.51
3	SRAS (mean, SD) [range of 0–14]	7.6 (4.3)	10.5 (4.7)	−2.42 (18)	0.03*
4	ATAQ (mean, SD) [range of 19–76]				
	Retaliation belief	9.6 (4.4)	7.6 (3.8)	1.83 (18)	0.08
	General belief	23.2 (6.3)	20.7 (6.4)	1.29 (18)	0.21
	Coping strategies	16.7 (3.2)	20.1 (4.2)	−2.64 (18)	0.02*
5	SCAS (mean, SD) [range of 0–28]	13.6 (6.4)	13.7 (5.3)	−0.04 (18)	0.97

One student dropped out

*TRAB* Teacher rating of aggressive behaviours (total maximum score = 42), *SDQ* Strengths and Difficulties Questionnaire (total maximum score = 40), *SRAS* Self rated aggression scale (total maximum score = 14), *ATAQ* Attitude towards aggression questionnaire (total maximum score = 76), *SCAS* Social cognition and attribution scale (total maximum score = 28)

* Data was statistically restricted with Bonferonni adjustment

A comparison of the post intervention scores of both groups reveal significant differences on the TRAB; Conduct sub-scale of the SDQ; as well as the SRAS. The intervention group had significantly lower scores post-intervention, as compared to the control group on the TRAB (t = −3.61, df = 35, p = 0.001), and on the SRAS (t = −2.80, df = 35, p = 0.008). However, the intervention group scored higher than the control group on the post-treatment Conduct sub scale of the SDQ (t = −2.37, df = 35, p = 0.02). See Table 4.

**Table 4:** Comparison of post intervention scores on outcome measures

No	Variable	Treatment group (N = 18)	Control group (N = 19)	t test t (df)	p value
1	Total TRAB (mean, SD) [range of 0–42]	18.22 (8.60)	26.47 (4.89)	−3.61 (35)	0.001*
2	SDQ (mean, SD)				
	Emotional	4.56 (1.72)	5.74 (4.94)	0.96 (35)	0.34
	Conduct	6.00 (3.43)	3.95 (1.51)	−2.39 (35)	0.02*
	Hyperactivity	7.61 (4.93)	5.79 (2.02)	−1.49 (35)	0.15
	Peer problems	6.28 (2.68)	6.89 (3.16)	0.64 (35)	0.53
	Total difficulties score [range of 0–40]	24.44 (7.78)	22.37 (7.51)	−0.83 (35)	0.41
	Prosocial	6.83 (3.94)	6.74 (1.37)	−0.10 (35)	0.92
3	SRAS (mean, SD) [range of 0–14]	6.11 (4.90)	10.53 (4.71)	−2.80 (35)	0.008*
4	ATAQ (mean, SD) [range of 19–76]				
	Retaliation belief	5.78 (2.67)	7.63 (3.76)	1.72 (35)	0.94
	General belief	17.17 (5.48)	20.74 (6.38)	1.82 (35)	0.08
	Coping strategies	22.11 (2.83)	20.05 (4.25)	−1.73 (35)	0.09
5	SCAS (mean, SD) [range of 0–28]	13.83 (5.26)	13.68 (5.68)	0.08 (35)	0.94

*TRAB* Teacher rating of aggressive behaviours (total maximum score = 42), *SDQ* Strengths and Difficulties Questionnaire (total maximum score = 40), *SRAS* Self rated aggression scale (total maximum score = 14), *ATAQ* Attitude towards aggression questionnaire (total maximum score = 76), *SCAS* Social cognition and attribution scale (total maximum score = 28)

* Data was statistically restricted with Bonferonni adjustment

Further analysis with ANCOVA showed statistically significant differences in the post-treatment scores on teacher rated aggressive behavior (TRAB) and self-rated aggression scale (SRAS) when controlled for their respective pre-treatment scores. For both measures, the intervention group scored significantly lower on aggression than the control group [TRAB {F (1, 34) = 11. 3, p = 0.002, (Cohen’s effect size (d) = 1.2}], and [SRAS {F (1, 35) = 11. 4, p = 0.002 (Cohen’s effect size (d) = 0.9}]. TRAB and SRAS each accounted for 25 % of the variance in the respective post intervention scores in the ANCOVA models. The assumption of homogeneity of regression slopes was met as evidenced by the absence of significant interactions. Inclusion of age in the model had no significant effect. The post intervention TRAB and SRAS scores between the two groups differed by more than one standard deviation each. The SDQ conduct scale which was higher in the intervention group at baseline remained higher post intervention. ANCOVA showed no treatment effect on the SDQ conduct Scale {F (1,34) = 1.61, p = 0.21} and the pre-intervention score was the only significant predictor of the post intervention SDQ conduct score {F(1,34) = 11.52, p = 0.002}. The other outcome measures were not significantly different post-intervention (Table 5).

**Table 5:** Analysis of co-variance (ANCOVA) results and effect sizes

No	Variable	F	p	Partial eta squared	Effect size (Cohen’s d)
1	TRAB scores	11.3	0.002*	0.247	1.2
2	SRAS	11.4	0.002*	0.251	0.9
3	SDQ conduct sub scale	1.6	0.213	0.045	–

* Data was statistically restricted with Bonferonni adjustment

### Impact of the intervention on teachers

At baseline, the 16 teachers were able to list an average of six possible triggers of aggressive behaviours in students. This list increased after the intervention to an average of 14; and significantly now included psychological triggers such as low self-esteem. The number of suggested strategies for reducing aggressive behaviours, by the teachers also increased from seven at baseline to 19 post-intervention. Incidentally, use of physical discipline was the most commonly suggested strategy (13 of the 16 teachers). Whereas only three teachers viewed psychological intervention as useful in managing students’ aggression at baseline, this increased to nine teachers post intervention.

## Discussion

This controlled intervention of the effectiveness of problem-solving skills for reducing aggressive behaviour in primary school children in Nigeria found significantly reduced teacher and self-rated aggression in the intervention group. Despite the short duration of the intervention and small sample, the study showed large effect sizes in these two outcome measures. To our knowledge, this is the first study of its kind in sub-Saharan Africa.

These findings are consistent with similar interventions from developed countries. For example, a systematic review by Glancy and Saini on psychological interventions for children with aggression and anger problems reported effect sizes ranging from 0.64 to 1.16 [36]. Another systematic review of school-based psychological interventions for aggressive behaviors also reported a mean effect size from 47 studies of 0.26 (range −0.71 to 1.29). The majority (60 %) of the studies had a positive effect size that was statistically significant [8]. The effect sizes for the teacher rated aggressive behaviours (TRAB) and the self rated aggression scale (SRAS) were quite large, at 1.2 and 0.9 respectively.

However, the current intervention did not show evidence of significant treatment effect on some of the outcome measures such as the SDQ, students attitude to aggression (ATAQ) and social cognition and attribution scale (SCAS). While there was a reduction in the mean SCAS scores in the intervention group from baseline {Mean 16.22 (SD = 6.37)}, to post intervention {Mean = 13.83 (SD = 5.26)}; the score for the control group increased {Baseline (Mean = 13.63 (SD = 6.44), post intervention (Mean = 13.68 (SD = 5.68)} but the differences were not statistically significant. Plausible reasons for this include the relatively short duration of the intervention (3 weeks) and small sample size (possible Type II Error). A previous systematic review found that interventions for aggressive behaviours that last 6 weeks or longer tend to have higher effect sizes than shorter ones [8]. For example, The Brain Power Program, which used similar attribution retraining strategies as the current study used 12 sessions [6]. Specifically, for the SDQ, some of the sub-scales (excluding the conduct subscale) had increased scores post intervention for both intervention and control groups. It is not exactly clear as to what may have accounted for this finding, but it may possibly be due to heightened awareness of their emotional, hyperactivity and peer relationship problems, following exposure to the measures at baseline.

Another significant aspect of this study is the impact on teachers. By observing the sessions unobtrusively and talking to the researchers after sessions, the teachers’ understanding of triggers of aggressive behaviours, range of strategies for managing these difficulties, and the potential role for psychological intervention improved. This change in the teachers’ perception and understanding despite not being directly targeted by the program suggests a possible role for this professional group in scaling up the delivery of behavioural programmes for aggressive children in Nigerian schools. This is particularly significant given the severe shortage of mental health professionals in this setting.

The positive treatment effects noted on the teacher and self-rated aggression scales after a relatively short intervention (6 sessions over 3 weeks) are promising, but it would require confirmation with further studies using independently rated assessments of changes in actual aggressive behaviours. A follow up study will also be required to explore the sustainability of the intervention benefits in the medium to longer term. It is important to note that the most effective evidence-based intervention for childhood aggression is parent management training (PMT) [37]. Thus future studies in Nigeria would benefit from exploring a dual synergistic intervention of problem solving skills training in schools alongside parent management training. However, given the potentially huge cultural and logistical challenge of running parenting programmes in Nigeria, it may be pragmatically more useful to focus initially on expanding school-based interventions as the school environment provides a ready and more easily accessible platform for such programmes which could potentially be delivered by teachers.

While there is the possibility of some teachers being unwilling to change their disciplinary behaviours from using corporal punishment to the more challenging use of this type of interventions, it is to be hoped that positive outcomes and engagements should convince them. Another potential barrier that will need to be surmounted, include the paucity of mental health professionals to deliver trainings, and provide support for teachers to deliver similar interventions. In the event that such teacher-led interventions are also effective, many more professionals will be required to scale up the intervention, but this will be a welcome problem to have.

## Limitations

While the findings of this study are promising, they should be interpreted in the light of some limitations. First, the students were not individually randomly allocated to treatment or control groups. Secondly, the small sample size and relatively short duration of the intervention may explain why no treatment effect was observed in some of the outcome measures. The study was powered to identify differences of one or more standard deviations; hence small differences, which may nonetheless be clinically important, may have been missed. Third, the absence of follow up data means we are unable to comment on the sustainability of the reported benefits. Fourth, the use of a wait-list control is known to be associated with higher effect sizes compared with active control groups. Fifth, the outcome measures were based on teacher and self-ratings rather than independently observed changes in behaviours; hence it is possible that socially desirable responding may explain some of the positive findings. Finally, given that the study was conducted in a high-density urban center in South West Nigeria, using only one school in each arm, the findings may not generalize to all schools in other urban or rural areas of Nigeria or other parts of Africa.

## Conclusions

School-based psychological interventions for reducing aggressive behaviour among primary school students in this environment appear feasible; and show promising effectiveness. The school setting provides a convenient platform for the introduction of such programs in order to reach the greatest number of children. There could be a potential role for teachers in implementing the programme in schools. This would help to integrate behavioural management programmes into the education ethos in Nigeria and improve its sustainability.

## Abbreviations

TRAB: teacher rated aggressive behaviour
SRAS: self-rated aggression scale
SDQ: strengths and difficulties questionnaire
ATAQ: attitude towards aggression questionnaire
SCAS: social cognition and attribution scale
WHO: The World Health Organization
USA: United States of America
LMICs: low and middle income countries
NER: net enrollment ratio
ANCOVA: analysis of covariance
